# Factors Associated With Access to and Timing of Coronavirus Testing Among US Adults After Onset of Febrile Illness

**DOI:** 10.1001/jamanetworkopen.2021.8500

**Published:** 2021-05-03

**Authors:** Mark J. Pletcher, Jeffrey E. Olgin, Noah D. Peyser, Madelaine Faulkner Modrow, Feng Lin, Jeffrey Martin, Thomas Carton, Alexis L. Beatty, Eric Vittinghoff, Gregory M. Marcus

**Affiliations:** 1 Department of Epidemiology and Biostatistics, University of California, San Francisco, San Francisco; 2 Division of General Internal Medicine, Department of Medicine, University of California, San Francisco, San Francisco; 3 Division of Cardiology, Department of Medicine, University of California, San Francisco, San Francisco; 4 Louisiana Public Health Institute, New Orleans

## Abstract

**Importance:**

Active SARS-CoV-2 (coronavirus) transmission continues in the US. It is unclear whether better access to coronavirus testing and more consistent use of testing could substantially reduce transmission.

**Objective:**

To describe coronavirus testing in persons with new onset of febrile illness and analyze whether there are changes over time and differences by race and ethnicity.

**Design, Setting, and Participants:**

This cohort study used data from the COVID-19 Citizen Science Study, launched in March 2020, which recruited participants via press release, word-of-mouth, and partner organizations. Participants completed daily surveys about COVID-19 symptoms and weekly surveys about coronavirus testing. All adults (aged at least 18 years) with a smartphone were eligible to join. For this analysis, US participants with new onset of febrile illness from April 2020 to October 2020 were included. Data analysis was performed from November 2020 to March 2021.

**Main Outcomes and Measures:**

Receipt of a coronavirus test result within 7 days of febrile illness onset.

**Results:**

Of the 2679 participants included in this analysis, the mean (SD) age was 46.3 (13.4) years, 1983 were female (74%), 2017 were college educated (75%), and a total of 3865 distinct new febrile illness episodes were reported (300 episodes [7.8%] from Hispanic participants, 71 episodes [1.8%] from Black participants, and 3494 episodes [90.4%] from not Black, not Hispanic participants) between April 2 and October 23, 2020. In weekly surveys delivered during the 14 days after fever onset, 12% overall (753 participants) indicated receipt of a test result. Using serial survey responses and parametric time-to-event modeling, it was estimated that by 7 days after onset of febrile illness, a total of 20.5% (95% CI, 19.1%-22.0%) had received a test result. This proportion increased from 9.8% (95% CI, 7.5%-12.0%) early in the epidemic to 24.1% (95% CI, 21.5%-26.7%) at the end of July, but testing rates did not substantially improve since then, increasing to 25.9% (95% CI; 21.6%-30.3%) in late October at the start of the winter surge. Black participants reported receiving a test result about half as often as others (7% [7 of 103] of survey responses vs 12% [53 of 461] for Hispanic vs 13% [693 of 5516] for not Black, not Hispanic; *P* = .03). This association was not statistically significant in adjusted time-to-event models (hazard ratio = 0.59 vs not Black, not Hispanic participants; 95% CI, 0.26-1.34).

**Conclusions and Relevance:**

Systematic underuse of coronavirus testing was observed in this cohort study through late October 2020, at the beginning of the winter COVID-19 surge, which may have contributed to preventable coronavirus transmission.

## Key Points

**Question:**

How often do persons with new febrile illness access coronavirus testing and receive a test result within 7 days of illness onset?

**Findings:**

In this cohort study, generally low rates of coronavirus testing were observed in 2679 participants reporting new onset of febrile illness. Although testing rates improved somewhat during the study period, timely coronavirus test results were sought and received by only 25.9% of newly febrile persons at the end of the study analysis period in late October 2020.

**Meaning:**

Our results suggest systematic underuse of coronavirus testing in patients with febrile illness that may contribute to community transmission.

## Teaser

This cohort study examines rates of testing for SARS-CoV-2 and receipt of test results within 7 days of febrile illness onset using data from smartphone-based weekly surveys administered as part of the COVID-19 Citizen Science Study.

## Introduction

The US has had a disproportionate share of morbidity and mortality from the COVID-19 pandemic.^1^ One potential explanation for this failure is the slow dissemination of testing capacity,^2^ but increases in testing rates through Fall 2020 did not seem to blunt the large COVID-19 surge that occurred the following winter.^3^

The effectiveness of coronavirus testing depends not only on the number of tests performed but also on who is getting tested. Testing may be administered for a variety of reasons: to diagnose COVID-19 in symptomatic patients, to detect asymptomatic infection in persons exposed to infection or at high risk of transmitting infection to others (eg, essential workers), to detect outbreaks in schools or other venues where in-person gatherings are now being allowed, or for other reasons that may be more or less useful for prevention of coronavirus transmission. Without knowing who is getting tested, it is unclear how effective coronavirus testing programs are in the US and, in particular, how available testing is to persons at highest risk for infection.

Persons with new onset of febrile illness are at relatively high risk of being infected with SARS-CoV-2 and infecting others. Fever is a cardinal sign of coronavirus infection,^4^ and appears relatively early in the course of disease.^5^ Therefore, it is of presumably high value to detect infection in newly febrile persons so they can isolate themselves, and so their contacts can get tested and quarantine themselves to avoid infecting others. It is not clear how effective current testing programs are at providing timely access to coronavirus testing in persons with new onset of febrile illness, and whether disparities in access to testing may be contributing to higher rates of COVID-19 illness and death in Black and Hispanic persons.^6,7^

The COVID-19 Citizen Science Study,^8^ launched in late March 2020, collects survey data daily on symptoms of COVID-19 and weekly on coronavirus testing. We used these responses to estimate time to receipt of test results among participants with new onset of febrile illness, and analyzed time trends in timely coronavirus testing over the course of the pandemic and disparities by race/ethnicity.

## Methods

### Study Design and Participants

The COVID-19 Citizen Science Study is a dynamic cohort study hosted on the National Institutes of Health–funded Eureka Research Platform, and delivered entirely via smartphone app. All adults with a smartphone who confirmed their phone number by text message were eligible and provided informed consent electronically before participating. There are no monetary incentives or other payments provided to participants. The study launched in late March 2020, and recruited primarily via press release, word-of-mouth, and partner organizations. For this analysis, we included only US adults who reported new onset of a febrile illness during follow-up through October 23, 2020. The study was approved by the University of California San Francisco Institutional Review Board.

### New Onset of Febrile Illness

We defined new onset of febrile illness as a report of either (1) fever or chills or (2) a temperature greater than 100.4 °F or 38.0 °C on a daily survey for participants enrolled for at least 5 days and having no prior report of fever in the prior 5 days. Participants with prior positive coronavirus test results were excluded. All episodes meeting entrance criteria, sometimes more than 1 per participant, were analyzed.

### Obtaining a Test and Receiving Results

All participants receive a weekly survey that included questions about coronavirus testing in the past week (without specifying a date). Survey questions were designed to distinguish tests for active infection (virus) or past infection (antibody); we assumed it was a test for active infection if participants were unsure. Focusing on tests for active infection, we coded each survey response as not tested, tested but awaiting results, tested with inconclusive results, or test results received (either positive or negative). Sequential survey responses (up to 3 received within 14 days) were used to specify the bounds of the interval during which a test was (or was not) received.

### Race, Ethnicity, and Other Participant Characteristics

Participants register with their date of birth, provide informed consent, and then complete a series of baseline surveys, including self-report of race (multiple categories allowed) and ethnicity. We categorized participants as “Hispanic, any race,” “Black, not Hispanic,” or “Not Hispanic, not Black,” according to their survey responses, given higher rates of COVID-19 illness and death in Black and Hispanic persons.^6,7^ Participants also reported subjective social status (MacArthur 10-point scale^9^) and education; use of cigarettes, e-cigarettes, and alcohol; and a variety of medical conditions. Current use was defined as any use in the last 30 days for cigarettes or e-cigarettes, and in the last week for alcohol.

### Statistical Analysis

We compared baseline characteristics across the 3 race/ethnicity categories for participants with at least 1 new febrile illness. For statistical testing across these 3 categories, we used χ^2^ or Fisher exact tests (when expected values were less than 5), and analysis of variance or Kruskal-Wallis tests (when outcomes were not normally distributed) for continuous variables. All tests were 2-sided, and the significance threshold was *P* < .05.

We then described weekly survey results about coronavirus testing, overall and stratified by race/ethnicity, subjective social status, days after illness onset that the weekly survey was delivered, and calendar month. Across these strata we compared response proportions, and then survey responses among respondents, using χ^2^ tests.

Because the exact date of testing was unknown and could be interval- or right-censored, we used a parametric Weibull model for estimating time to receipt of a test result. To define the interval during which a test result was received, we used up to 3 weekly survey results reported on the day of or up to 14 days after onset of febrile illness. Survey results received after illness day 7 were ignored if the participant did not respond to a previous survey delivered on or before day 7 (making subsequent surveys about the last 7 days difficult to interpret). Fitted cumulative incidence of testing was plotted along with survey responses by days after onset of illness. All models accounted for clustering and nonindependence due to multiple episodes per person.

To analyze changes in testing availability during different phases of the pandemic, we flexibly modeled calendar time using a cubic spline with 3 knots, and present tests for time trend overall and for nonlinearity. We then tested the contribution of our 3-level race/ethnicity variable to that model by including a main effect (with 3 levels) and interactions with each of the time trend spline variables. The main effect was also estimated in models adjusting for age, sex, medical conditions, cigarette/e-cigarette use and alcohol use, and then additionally for subjective social status and education.

We conducted sensitivity analyses to explore how our results might change with different assumptions about missing survey responses. In the first, we assumed that a test result was received 1 day after a participant reported that they were awaiting a test result, unless we know from a subsequent survey response that such a result was definitely not received. In more extreme sensitivity analyses, we assumed that any person who did not respond to a survey about coronavirus testing had actually not received coronavirus testing (one extreme), or had actually received a test result on day 3 after onset of febrile illness if their first survey response was missing or 1 day after their last completed response (the other extreme). We also conducted 3 analyses, under base case assumptions, exploring results in 3 subsets of episodes: (1) the first episode for each person; (2) episodes where fever was reported on both the first and the second day; and (3) episodes where a high temperature (>100.4 °F or 38.0 °C) was reported (rather than just subjective “fever or chills”).

We used Stata statistical software version 16.1 (StataCorp) for all analyses. Parametric interval-censored survival-time regression was accomplished using Stata’s stintreg command. Data analyses were performed from November 2020 to March 2021.

## Results

Between March 26 and October 23, 2020, 37 436 people enrolled in the COVID-19 Citizen Science Study; 2% self-identified as Black and 7% as Hispanic. After enrollment, 3001 participants reported at least one episode of fever or chills or a high temperature and met our criteria for new onset of a febrile illness. We excluded 95 with incomplete data on race or ethnicity, and 227 who live outside the US. The 2679 participants included in our analysis contributed a total of 3865 episodes overall, including 300 episodes (7.8%) from 183 Hispanic participants, 71 episodes (1.8%) from 41 Black participants, and 3494 episodes (90.4%) from 2455 participants who were neither Black nor Hispanic.

Among the 2679 participants included in our analysis with at least 1 febrile episode, age varied widely with a mean (SD) of 46.3 (13.4) years, most were female (n = 1983; 74%) and college-educated (n = 2017; 75%), and few reported comorbid illness (eg, 656 participants with hypertension [24%] and 176 participants with diabetes [6.6%]) (Table 1). Few participants reported current cigarette use (n = 164 [6.1%]), but more than half reported current alcohol use (n = 1399 [52%]).

**Table 1.** Characteristics of COVID-19 Citizen Science Participants Developing at Least One New Febrile Illness

Baseline characteristics	Race/ethnicity, No. (%)			*P* value^a^
	Hispanic, any race	Black, not Hispanic	Not Hispanic, not Black	
Participants, No.	183	41	2455	NA
Episodes, No.	300	71	3494	NA
Age, y				
18-29	30 (16)	7 (17)	208 (8)	.01
30-39	45 (25)	9 (22)	657 (27)	
40-49	45 (24)	5 (12)	588 (24)	
50-59	41 (22)	12 (29)	561 (23)	
≥60	22 (12)	8 (20)	441 (18)	
Sex				
Female	135 (74)	31 (76)	1817 (74)	.33
Male	48 (26)	9 (22)	630 (26)	
Prefer not to disclose	0	1 (2)	8 (0.3)	
Self-reported race				
American Indian or Alaskan Native	4 (2)	2 (5)	25 (1)	.03
Asian	8 (4)	2 (5)	131 (5)	.93
Black	7 (4)	41 (100)	0	<.001
Native Hawaiian or Pacific Islander	3 (2)	0	6 (0.2)	.04
White	143 (78)	11 (27)	2339 (95)	<.001
Don’t know	2 (1)	0	5 (0.2)	.11
Subjective socioeconomic status^b^				
Mean (SD)	6.1 (1.8)	5.9 (1.8)	6.6 (1.7)	<.001
<6	68 (37)	12 (29)	600 (24)	.001
6-8	101 (55)	29 (71)	1601 (65)	
9-10	14 (8)	0	254 (10)	
Education				
High school degree or less	6 (3)	3 (7)	92 (4)	<.001
Some college	64 (35)	9 (22)	452 (18)	
College graduate	55 (30)	13 (32)	841 (34)	
Post-graduate	57 (31)	15 (37)	1036 (42)	
Other or missing	1 (0.6)	1 (2)	34 (1)	
Current cigarette use^c^	13 (7)	4 (10)	147 (6)	.40
Current e-cigarette use^c^	7 (4)	0	90 (4)	.61
Current alcohol use^c^	79 (63)	17 (63)	1303 (67)	.52
High blood pressure or hypertension^c^	46 (25)	14 (34)	596 (24)	.34
Diabetes^c^	15 (8)	0	161 (7)	.14
Heart disease^c^	20 (11)	10 (24)	165 (7)	<.001
Stroke^c^	3 (2)	3 (7)	54 (2)	.10
COPD^c^	11 (6)	4 (10)	69 (3)	.004
Cancer undergoing active treatment^c^	8 (4)	3 (7)	104 (4)	.49
Immunodeficiency^c^	9 (5)	5 (14)	99 (4)	.03
Pregnant^c^	3 (2)	0	13 (0.5)	.15
Total No. of febrile episodes during follow-up				
Median (IQR)	1 (1-2)	1 (1-2)	1 (1-1)	.001
Mean (SD)	1.6 (1.2)	1.7 (1.0)	1.4 (1.0)	.005

Abbreviations: COPD, chronic obstructive pulmonary disease; IQR, interquartile range; NA, not applicable.

^a^ *P* values calculated using a χ^2^ test, Fisher exact test, one-way analysis of variance or Kruskal-Wallis tests (see Methods section). For education, we used a χ^2^ test excluding the other/missing category.

^b^ Subjective social status was self-reported using the MacArthur 10-point scale,^9^ with 10 being the highest.

^c^ Some participants were missing measurements, refused to answer, or answered “don’t know” for cigarette use (n = 26), e-cigarette use (n = 20), alcohol (n = 588), high blood pressure (n = 13), diabetes (n = 12), heart disease (n = 5), stroke (n = 22), COPD (n = 24), cancer (n = 10), immunodeficiency (n = 61), pregnant (n = 18).

Even with small numbers of Black and Hispanic participants, some systematic differences were noted across the 3 groups, including lower levels of education and subjective social status in Black and Hispanic participants, and higher prevalence of some medical conditions in Black participants. Black and Hispanic participants contributed more febrile episodes each than participants who were neither Black nor Hispanic (Table 1).

**Figure 1.**
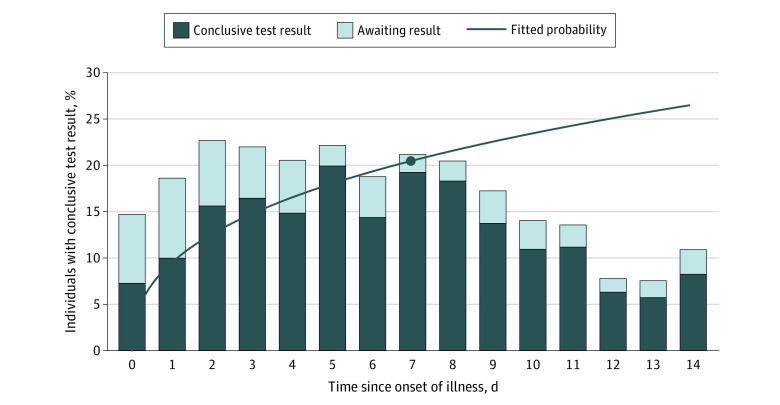
Self-reported Coronavirus Test Result Status as Reported by Participants With New Febrile Illness by Days Since Onset Fitted probability represents the cumulative proportion with a coronavirus test result by days after onset of illness estimated with an unadjusted interval-censored Weibull model (see Methods section) using all survey responses. At 7 days after onset, the model estimated a cumulative proportion of 20.5% (95% CI, 19.1%-22.0%) (circular data marker). This overall result does not consider calendar date or other covariates.

A total of 8006 weekly surveys about coronavirus testing were delivered up to 14 days after fever onset (1-3 surveys/episode), with a 76% response rate (n = 6080 total responses). Across all survey responses, 5051 participants (83%) reported they had not been tested, and only 753 participants (12%) noted receiving a test result; an additional 276 participants (5%) noted that they had taken a test but not yet received a test result (Table 2). For surveys completed on day 0 (the same day that a fever was first reported), 7% (92 participants) reported already having received test results; this proportion increased steadily over the course of illness to a peak of 20% (76 participants) on day 5 after onset (Table 2, Figure 1, *P* < .001).

**Table 2.** Response to Weekly Survey Questions About Testing up to 14 Days After a Febrile Illness

Characteristic	Episodes, No.	Weekly surveys delivered in first 2 weeks after new onset of febrile illness reported		Survey response, No. (% of responses)		
		Delivered, No.	Responded, No. (% of delivered)	Not tested	Tested	
					Awaiting results^a^	Received a test result (positive or negative)
Total	3865	8006	6080 (76)	83	5	12
Race/ethnicity						
Hispanic, any race	300	644	461 (72)	82	7	12
Black, not Hispanic	71	149	103 (69)	90	3	7
Not Hispanic, not Black	3494	7213	5516 (76)	83	4	13
*P* value	NA	NA	.003^b^	NA	NA	.03^c^
Subjective social status						
<6	1107	2325	1696 (73)	84	5	11
6-8	2417	4979	3837 (77)	83	5	13
9-10	341	702	547 (78)	82	4	14
*P* value	NA	NA	<.001^b^	NA	NA	.33^c^
Days after illness onset						
0	NA	1371	1257 (92)	85	7	7
1	NA	378	348 (92)	81	9	10
2	NA	460	357 (78)	77	7	16
3	NA	478	363 (76)	78	6	17
4	NA	473	369 (78)	79	6	15
5	NA	518	382 (74)	78	2	20
6	NA	483	361 (75)	81	4	14
7	NA	671	519 (77)	79	2	19
8	NA	559	311 (56)	79	2	18
9	NA	408	283 (69)	83	4	14
10	NA	398	270 (68)	86	3	11
11	NA	416	294 (71)	86	2	11
12	NA	409	281 (69)	92	1	6
13	NA	401	275 (69)	92	2	6
14	NA	583	410 (70)	89	3	8
*P* value	NA	NA	<.001^b^	NA	NA	<.001^c^
Month						
April	588	1278	978 (77)	91	3	6
May	592	1279	949 (74)	87	4	9
June	448	948	675 (71)	81	5	13
July	494	1043	771 (74)	78	7	14
August	519	1098	840 (77)	82	4	14
September	694	1467	1160 (79)	82	4	14
October	530	893	707 (79)	77	6	18
*P* value	NA	NA	<.001^b^	NA	NA	<.001^c^

Abbreviation: NA, not applicable.

^a^ Includes 7 inconclusive test results.

^b^ *P* values are from a χ^2^ test comparing response (responded or not) across categories.

^c^ *P* values are from a χ^2^ test comparing the 3-level survey response variable across categories, among respondents.

**Figure 2.**
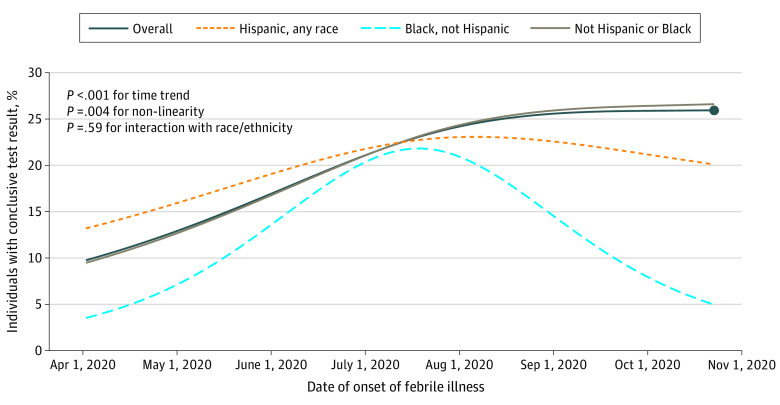
Proportion With a Coronavirus Test Result at 7 Days After Febrile Illness Onset, Over Calendar Time, by Race/Ethnicity Proportions represent fitted cumulative hazard estimates from interval-censored Weibull models (see Methods section) that include date (fit as a cubic spline), with and without race/ethnicity also included (main effect and interactions with spline variables). On October 23, 2020 (the last episode date analyzed), the model estimated that overall 25.9% (95% CI, 21.6%-30.3%) of persons with a febrile illness will get a test result by 7 days after onset of their illness (circular data marker). With interactions included, estimates on October 23, 2020, were 5% (95% CI, −8% to 18%) for Black, not Hispanic participants; 20% (95% CI, 4%-36%) for Hispanic, not Black participants; and 27% (95% CI, 22%-31%) for participants who were not Hispanic or Black.

Accounting for all survey responses submitted during the course of illness, we estimate that 20.5% (95% CI, 19.1%-22.0%) of patients received a coronavirus test result within 7 days of febrile illness onset (Figure 1). Substantial changes were apparent over the course of the pandemic (Figure 2, *P* < .001 for time trend); coronavirus test results at 7 days after febrile illness increased from 9.8% (95% CI, 7.5%-12.0%) at the beginning of April to 24.1% (95% CI, 21.5%-26.7%) by the end of July. Progress slowed at that point (*P* = .004 for nonlinearity); by late October, coronavirus testing within 7 days had increased only slightly more, to 25.9% (95%CI, 21.6%-30.3%) (Figure 2).

Black participants reported receiving a test result only about half as often as other participants (7% of responses in Black participants [7/103] vs 12% of responses in Hispanic participants [53/461] vs 13% of responses in participants who were not Black and not Hispanic [693/5516]; *P* = .03) (Table 2). This association was also apparent in statistical models but was not statistically significant in adjusted models (Table 3).

**Table 3.** Relative Likelihood of Receiving a Coronavirus Test Result

Race/ethnicity	Receipt of coronavirus test results in the US, hazard ratio (95% CI)		
	Unadjusted^a^	Adjusted for age, sex, medical conditions, cigarette/e-cigarette and alcohol use	Additionally adjusted for subjective social status and education
Hispanic, any race	1.01 (0.73-1.40)	0.99 (0.73-1.37)	1.02 (0.74-1.40)
Black, not Hispanic	0.55 (0.24-1.23)	0.60 (0.27-1.34)	0.59 (0.26-1.34)
Not Hispanic, not Black	1 [Reference]	1 [Reference]	1 [Reference]
*P* value^b^	.34	.46	.45

^a^ All results include calendar time, modeled as a cubic spline.

^b^ *P* value represents a test of the 3-category race/ethnicity variable, main effect only.

In a sensitivity analysis, we assumed that a test result was received 1 day after a participant reported that they were awaiting a test result (unless we know otherwise from a subsequent survey response), and found a slightly higher proportion receiving timely test results (26.8%; 95% CI, 22.4%-31.1%). In another, we assumed nonrespondents actually received a coronavirus test on day 3 of illness (or 1 day after their last completed response). Under these extreme assumptions, timely testing rates were higher (43.9%; 95% CI, 39.3%-48.5%) but remained substantially below 50%. With assumptions set at the other extreme (nonresponse means no test performed), timely testing rates were lower (22.4%; 95% CI, 18.6%-26.2%). We also analyzed results, with base case assumptions, in several subsets of febrile episodes: Timely testing rates were 30.0% (23.0%-35.6%) when limiting to first episodes per participant, 34.2% (95% CI, 24.7%-43.7%) in the subset where fever was reported also on the second day of illness, and 34.7% (95% CI, 22.5%-47.0%) in the subset where a high temperature greater than 100.4 °F or 38.0 °C was reported.

## Discussion

In this analysis of the COVID-19 Citizen Science Study, we found low rates of coronavirus testing after new onset of a febrile illness. We estimate that in late October 2020, when this analysis was completed, only 25.9% of persons with new febrile illness were receiving a test result within 7 days. We found that Black participants were about half as likely to report receiving test results than other participants; but with only limited numbers of such participants in our study, we could not make conclusions about this with statistical certainty. Improvements in testing rates after onset of febrile illness were apparent early in the pandemic, but very little improvement occurred after testing ramped up in Summer 2020.

Testing capacity in the US is known to have been limited early in the pandemic.^2,10,11^ Our models indicate that only 9.8% of persons with new onset of febrile illness at the beginning of April 2020 received a test result within a week. With the Food and Drug Administration’s issuance of Emergency Use Authorizations for medical devices related to coronavirus testing,^12^ the National Institute of Health’s Rapid Acceleration of Diagnostics (RADx) Program^13^ and other federal efforts to support expansion of testing capacity,^14^ testing volume rapidly expanded through the end of July to nearly 1 million tests per day.^1^ This increase is mirrored in our data, which show testing by 7 days after febrile illness onset to have increased from 9.8% to about 24.1% in late July. National data from August through October show continuing (although slower) increases in testing volume in the US to approximately 1.2 million tests per day in late October.^1^ Despite this increase in testing volume, we saw very little continued increase in the likelihood of getting tested after a febrile illness, which we estimate at 25.9% at the time of our analysis in late October, such that nearly three-fourths of persons with febrile illness remain untested for coronavirus a week after onset of their illness. We are not aware of other data available for estimating testing rates in symptomatic persons or others for whom testing might be particularly important for disease control.

We sought to determine whether coronavirus testing disparities might be contributing to disparities in coronavirus outcomes, which are well documented.^6,7,15,16^ We found lower rates of testing in Black participants, but these differences were not statistically significant due to the small numbers of Black participants in our sample. Prior evidence on this point is mixed. Analyses have demonstrated lower testing rates in New York City neighborhoods with a lower proportion of White residents,^17^ and fewer testing sites and longer travel times to testing sites in areas with higher proportions of minority residents.^18,19^ Analyses of individual patients using medical records have demonstrated lower testing rates in non-English speakers in Washington state,^20^ but higher testing rates in Black patients than in either Hispanic or non-Hispanic White patients among patients receiving care at the US Department of Veterans Affairs^21^ and among active patients in 53 health systems across 21 states analyzed by the Kaiser Family Foundation.^22^ Unlike total testing volume and positivity rates, the race and ethnicity of patients tested for coronavirus are not widely available^23^ so population-level testing rates by race and ethnicity cannot be calculated using publicly reported data.

### Limitations

Our sample and analysis are subject to a number of limitations. Because of the small number of Black and Hispanic participants in our sample, we were underpowered to detect disparities in timely coronavirus testing after onset of febrile illness, so our lack of statistically significant differences by race and ethnicity should not be interpreted as absence of a disparity in testing in the population. Our sample also skews toward higher education and social status than the general population, as is typical for internet-based volunteer samples.^24^ However, this stratum of the US population generally has better access to health care and resources than others, so if testing rates are low in our participants, they are likely even lower in more vulnerable subsets of the population. Participants in our study are prompted to report symptoms on a daily basis, but they do not necessarily submit a response every day; we are likely to miss some febrile illnesses completely, and to catch some illnesses 1 or more days after onset. Evidence of this phenomenon can be seen in Table 2, which shows that substantially more survey responses are received on day 0 of febrile illness than would be expected, possibly due to the SMS (text) message participants receive with the weekly survey. The result of reporting a fever late (days after it truly started) would be to artificially inflate the proportion receiving a test result by 7 days after onset, so actual testing rates at 7 days are likely to be even lower than estimates from our analysis. We are missing some responses in our weekly testing surveys; but even extreme sensitivity analyses imputing test results when survey responses are missing yield estimates of testing at 7 days after onset of febrile illness that remain quite low compared with what might be optimal or expected. Finally, we are unable to determine why patients did not receive a coronavirus test.

## Conclusions

This cohort study’s results suggest systematic underuse of coronavirus testing in patients with febrile illness. Whether this is because of lack of testing availability, knowledge about how to get a test, understanding about the importance of testing, or active avoidance (eg, to avoid economic hardships associated with isolation and quarantine of contacts if one tests positive) is unclear. We cannot know for certain what the impact of more effective targeting of coronavirus testing would be on disease transmission in the US, how it might have blunted the 3rd wave that recently swept through the US,^25^ or how it might be used to reduce transmission of new coronavirus variants.^26^ However, it is clear that countries such as China^27^ and South Korea^28,29^ have a much more aggressive targeted approach to testing and appear to have substantially lower community transmission rates. Clear guidelines with well-resourced public health service announcements targeted to both clinicians and the public, ensuring adequate test capacity and convenience, and provision of resources to mitigate the hardships of isolation and quarantine that come with a positive test are all likely to reduce barriers to coronavirus testing and increase the likelihood of identifying new infections in the community and reducing transmission in the US.
